# Air pollution’s toll on adverse birth outcomes: key research insights from Chinese epidemiological studies

**DOI:** 10.3389/fpubh.2025.1616787

**Published:** 2025-07-25

**Authors:** Hui Chang, Wanting Sun, Yuanyuan Tian, Jing Wu, Yanru Yue, Wenhua Li, Xin Zhao, Xiaoan Zhang, Jian Jin

**Affiliations:** ^1^Department of Clinical Research and Translational Medicine, The Third Affiliated Hospital of Zhengzhou University, Zhengzhou, China; ^2^Tianjian Laboratory of Advanced Biomedical Sciences, School of Life Sciences, Zhengzhou University, Zhengzhou, China; ^3^School of Public Health, Xi'an Jiaotong University, Xi'an, China; ^4^Zhengzhou University, Zhengzhou, China; ^5^Department of Immunology, School of Basic Medical Science, Xinxiang Medical University, Xinxiang, China

**Keywords:** air pollution, birth outcome, preterm birth, stillbirth, spontaneous abortion

## Abstract

Despite extensive global evidence linking air pollution to adverse birth outcomes, a comprehensive synthesis of China-specific epidemiological insights—particularly across its regionally diverse pollution landscapes and susceptible populations—remains limited. This systematic review integrates evidence from Chinese epidemiological and retrospective studies (2011–2023) retrieved via PubMed, Web of Science, and CNKI, to address critical gaps in contextual understanding of how air pollution uniquely impacts birth outcomes in China. We identify robust associations between exposure to pollutant and risks of miscarriage, preterm birth, low birth weight, and birth defects in Chinese cohorts, while revealing understudied areas such as ozone, heavy metals, and gene–environment interactions. Crucially, our analysis demonstrates how regional policy intervention attenuated birth risks in high-exposure zones, offering actionable models for policymakers. To resolve mechanistic uncertainties, we propose integrating machine learning and multi-omics approaches in future research. This review provides the first China-focused roadmap for optimizing environmental regulations and prenatal protections, directly informing public health strategies tailored to its demographic and pollution realities.

## 1 Introduction

A country’s future and development depend on the health of its newborns, and the “14th Five-Year Plan” proposes to enhance newborn health through the maternal and child safety plan. In 2016, China formulated the Outline of the Healthy China 2030 Plan, which stipulates that by 2030, China’s infant mortality rate should be reduced to 5 per thousand, and the mortality rate for children under five and mothers should be reduced to 5 per thousand and 12 per 100,000. Global and age-specific health effects are associated with air pollution, a major environmental risk. The risk of preterm birth, stillbirth, low birth weight, and birth defects is increased when pregnant women are exposed to air pollution. Currently, there is a long way to go to combat air pollution. Thus, air pollution and adverse birth outcomes need to be studied urgently.

Atmospheric pollution refers to the phenomenon in which certain substances are emitted by human activities or natural processes, and persist for an extended period with high density, thereby causing harm to human health, comfort, welfare, or the environment (1). Any kind of substance that degrades the quality of the atmosphere can be called an air pollutant. Air pollutants mainly include gaseous pollutants, persistent organic pollutants, heavy metals, and particulate matter. Gaseous pollutants in the atmosphere are mainly caused by fuel combustion. The main sources of gaseous pollutants are combustion of fuels, such as NO_2_, SO_2_, and O_3_. In China, the primary type of air pollution consists of coal-smoke, characterized mainly by inhalable particles (PM_10_) and sulfur dioxide (SO_2_) (2). Human health is adversely affected by atmospheric pollution, causing nearly 7 million premature deaths each year and even more medical consultations and hospitalizations (3). The 2020 State of Global Air Report released by the U.S. Institute for Health Impact Research states that air pollution in 2019 led to the deaths of nearly half a million babies globally within the first month of life, with nearly two-thirds of those deaths linked to indoor air pollution (4). It is becoming increasingly apparent that atmospheric pollution poses health risks to specific groups, particularly pregnant women and fetuses. Specifically, for every 10 μg/m^3^ increase in PM_2.5_ concentration, there is an associated decrease of 22 g in birth weight, an 11% increase in the risk of low birth weight (LBW), and a 12% increase in the risk of preterm birth (PTB). Furthermore, the global population-weighted average birth weight decrease of 89 g in 2019 can be attributed to total PM_2.5_ exposure (5). Over the past few years, scholars at home and abroad have conducted numerous studies showing that exposure to air pollutants during pregnancy is potentially harmful to pregnant women and fetuses (6–8).

The term “adverse birth outcomes” (ABO) describes a range of pathological pregnancy and delivery issues that do not result in live births. These complications include low birth weight, macrosomia, congenital abnormalities, stillbirth, preterm birth, spontaneous abortion, fetal death, and other adverse pregnancy outcomes (9). In order to support and validate public health intervention efforts, this paper reviews the association involving air pollution and unfavorable birth outcomes, which has been demonstrated by epidemiologic and retrospective investigations.

While previous systematic reviews have established global associations between air pollution and adverse birth outcomes, critical gaps persist in contextualizing these risks within China’s unique sociodemographic, environmental, and policy landscape. This review provides the first comprehensive synthesis of China-exclusive epidemiological evidence (2011–2023) to deliver three key advances:

1. Quantification of region-specific pollutant-birth outcome relationships across China’s heterogeneous air quality zones.
2. Analysis of unappreciated interactions between pollution exposures and China-specific susceptibility factors (gene-nutrient-pollution triads in Han populations).
3. A mechanistic roadmap proposing machine learning and multi-omics integration to resolve causality gaps.

By translating China’s evidence into actionable public health strategies, this work addresses an urgent need for context-driven prenatal protections.

## 2 Methods

### 2.1 Literature search strategy

Databases: PubMed, Web of Science, CNKI, and China National Knowledge Infrastructure (Chinese studies). Timeframe: January 2011–December 2023, (reflecting China’s PM < 2.5 monitoring standardization), Search Terms: Customized syntax combining (“air pollution” OR “PM < 2.5”) + (“birth outcome” OR “preterm birth”) + (“China” OR “Chinese”). Inclusion/Exclusion Criteria: Population: Pregnant women in China. Exposure: Ambient air pollution, Outcomes: Miscarriage, preterm birth, low birth weight, birth defects. Study Types: Epidemiological/retrospective studies >500 subjects. Exclusion: Non-Chinese cohorts, reviews, non-English/Chinese papers.

### 2.2 Air pollution and preterm birth

PTB is defined by the World Health Organization as a delivery that occurs before 37 complete weeks (<259 days) of gestation, whereas very preterm birth (VPTB) is defined as a delivery that occurs between 28 and 32 weeks of gestation (9). It was reported that PTB continues to be the primary cause of death for children under the age of five (10). Furthermore, other issues such as lung infections, airway damage, eye disease, and neurological disorders are linked to PTB (11, 12). Although there are currently few studies on the relationship between air pollution and PTB-related disease incidence, some have found that particle matter in air pollutants significantly affects respiratory systems in PTB. Up to now, the correlation between air pollution and PTB is not consistent. Some studies have found that exposure to air pollutants is positively correlated with the risk of PTB, while others have not found significant associations between pollution and PTB.

Although several later studies have indicated that air pollution may have some effect on premature birth, research carried out in England between 2004 and 2008 (13) found no link between air pollution and PTB (14, 15). In particular, the study carried out in Chongqing, through the collection of meteorological factors, air pollutants, birth certificate system information and other data in 9 districts of Chongqing from 2015 to 2020. This demonstrates that there is a substantial relationship involving PTB and air pollution, and that while the cumulative effect grows with time, the relative risk declines (16). The inconsistencies of the conclusions may be influenced by research methods, environmental pollution degree, social and economic level, etc.

### 2.3 Correlation of particulate matter (PM) with preterm birth

In Wuhan, China, a population-based prospective birth cohort study comprising 95,911 live births between 2011 and 2013 revealed that the risk of postpartum bleeding increased by 3% on average every 5 years (*OR* = 1.03, 95%*CI*: 1.02–1.05) for every 5 μg/m^3^ increase in PM_2.5_ concentration, and by 2% on average every 5 years for every 5 μg/m^3^ increase in PM_10_ concentration (*OR* = 1.02, 95%*CI*: 1.02–1.03) (17). According to a study conducted in Zhejiang Province, there is a significant correlation between exposure to air pollutants and the risk of PTB. Specifically, there is a 17% increase in preterm birth risk for every 10 μg/m^3^ increase in PM10 exposure (*OR* = 1.17, 95%*CI*: 1.06–1.30) and a 13% increase in preterm birth risk for every 10 μg/m^3^ increase in PM_2.5_ exposure (*OR* = 1.13, 95%*CI*: 1.03–1.25) (18). A cohort study from Wuxi, Jiangsu Province, suggests that high levels of PM_10_ exposure during the second trimester increase the risk of preterm birth (*OR* = 1.42, 95%*CI*: 1.10–1.85) (19). Studies conducted in other regions have reached similar conclusions (20, 21). The *OR* value and 95%*CI* of the effect of different concentrations of particulate matter on preterm birth was shown in Table 1 for details.

**Table 1:** The *OR* value and 95%*CI* of the effect of different concentrations of particulate matter on preterm birth.

Pollutant concentrations	Air pollution	Analysis city	*OR*	Outcome	Risk increase rate	95%*CI*	Reference
5 μg/m^3^	PM_2.5_	Wuhan	1.03	Preterm birth	3%	1.02–1.05	Qian et al. (17)
	PM_10_	Wuhan	1.02	Preterm birth	2%	1.02–1.03	Qian et al. (17)
10 μg/m^3^	PM_2.5_	Zhejiang	1.13	Preterm birth	13%	1.06–1.30	Sun et al. (18)
	PM_10_	Zhejiang	1.17	Preterm birth	17%	1.03–1.25	Sun et al. (18)
10 μg/m^3^	NO_2_	Shanghai	1.07	preterm birth	7%	1.02–1.13	Ji et al. (22)
10 μg/m^3^	O_3_	Shiyan	1.029	Preterm Birth	2.9%	1.004–1.056	Li et al. (20)
10 μg/m^3^	SO_2_	Jingzhou	1.082	Preterm Birth	8.2%	1.023–1.144	Li et al. 2018 (20)
10 μg/m^3^	SO_2_	Shiyan	1.28	Preterm Birth	28%	1.04–1.39	Chen et al. (15)
100 μg/m^3^	CO	Wuhan	1.15	Preterm birth	15%	1.11–1.19	Ming et al. (16)
1.8 mg/m^3^	CO	Como	0.84	Preterm birth		0.72–0.99	Capobussi et al. (26)

### 2.4 Association of NO_2_ with preterm birth

Increased NO_2_ exposure was associated with preterm birth in a retrospective cohort study of 25,493 single pregnancies conducted during 2014–2015 at a major Shanghai maternity hospital. The risk of preterm birth increased by 3% (95%*CI*: 0.96–1.10) and 7% (95%*CI*: 1.02–1.13) for every 10 μg/m^3^ increase in NO_2_ exposure throughout and throughout the third trimester, respectively. One month and 1 week before delivery, the risk increased by 10% (95%*CI*: 1.04–1.15) and 5% (95%*CI*: 1.00–1.09), respectively. However, the risk of preterm birth was not significantly enhanced by increased NO_2_ exposure in the first and second trimesters (22).

Studies conducted in Jingzhou (20) and Changsha (23) have yielded comparable findings indicating that exposure to NO₂ during pregnancy increases preterm birth risk. Additionally, a meta-analysis published in 2022 reported similar findings (24).

### 2.5 Association of O_3_ with preterm birth

A US study that examined the electronic medical records of 5,0005 women who gave birth in 20 Utah hospitals between 2002 and 2010 discovered that the risk of postpartum bleeding in a subsequent pregnancy rose by 17% with greater O_3_ exposure levels than at consistently low exposure (25). A comparable study from Shiyan, China, observed a 2.9% increase in PTB risk (*OR* = 1.029, 95%*CI*: 1.004–1.056) for every 10 μg/m^3^ increase in O₃ exposure (20). Research from Zhejiang Province consistently demonstrated the following outcomes when utilizing the quasi-AQI model (AQI, or air quality index, which integrates numerous air pollution concentrations into one composite index) and principal component analysis—Generalized linear model (PCA-GLM): PTB risk is increased by exposure to O_3_ (14).

### 2.6 Association of SO_2_ with preterm birth

After adjusting for confounding variables, the Shiyan City study examined meteorological and atmospheric pollutant concentration data from 13,111 pregnant women who gave birth between 2015 and 2017. Cox regression analysis was then performed, revealing that the risk of VPTB increased by 28% (95%*CI*: 1.04–1.39) for every 10 μg/m^3^ increase in SO_2_ exposure in the first trimester (15). In the Jingzhou study, PTB risk increased by 8.2% (*OR* = 1.082, 95%*CI*: 1.023–1.144) for per 10 μg/m^3^ increase in SO₂ exposure (20). Meanwhile, research conducted in China and the USA revealed that prenatal exposure to SO_2_ has also been linked to a considerable risk of preterm delivery (25). However, the results of the study conducted in Como, Italy, from 2005 to 2012 showed that exposure to high levels of SO₂ (5.5 mg/m^3^) appeared to delay delivery. This may be because a longer gestation period could compensate for damage caused by maternal hypoxia (26).

### 2.7 Association of CO with preterm birth

The results of the limited research that has been done on the subject of CO and preterm delivery are not always consistent. The Utah study found that higher CO exposure levels, compared to prolonged low exposure, were associated with a 31% greater incidence of PTB in the second pregnancy (25). The Wuhan study also revealed that the risk of PTB increased by 15% every 5 years for every 100 μg/m^3^ increase in CO concentration (*OR* = 1.15, 95%*CI*: 1.11–1.19) (17). Similar findings have been achieved by studies carried out in other locations (18). Nonetheless, a study carried out in Como, Italy, revealed a negative correlation between high CO exposure (1.8 mg/m^3^) and the incidence of PTB (*aOR* = 0.84, *CI*: 0.72–0.99) (26). It’s possible that the varied dates, places, and demographics of the subjects contributed to the contradictory findings. In the meantime, a meta-analysis’s findings revealed that the combined relative risk of PTB was 1.032, 1.070, moderate PTB was 0.859, 1.081, very PTB was 1.119, 1.194, and high PTB was 1.128, 1.259 when the mother was exposed to PM_2.5_, PM_10_, NO_2_, O_3_, SO_2_, and CO during pregnancy (27).

A Korean study showed that air pollution increases the incidence of high-risk pregnancies (positively correlated with PM_10_, PM_2.5_, CO, NO_2_, SO_2_), with gestational diabetes ranking first, followed by preterm birth (28). A study conducted in Zhejiang Province analyzed the data using a logistic curve model and found that the exposure thresholds (lowest observed adverse effect levels) for PM_2.5_, PM_10_, SO_2_, NO_2_, CO and O_3_ in pregnant women were 44.3 μg/m^3^, 65.1 μg/m^3^, 18.5 μg/m^3^, 27.3 μg/m^3^, 2038.8 μg/m^3^ and 108.3 μg/m^3^, respectively. They were all below the federal guidelines for air quality and the environment (18). In conclusion, there is a correlation between air pollution and the risk of premature delivery. Therefore, it is important to monitor the air quality in one’s home throughout pregnancy in order to lower the risk of unfavorable pregnancy outcomes.

## 3 Effects of air pollution on stillbirth

A fetus is considered dead when it passes away after 20 weeks of gestation. One kind of prenatal death is stillbirth, which occurs when the fetus passes away during delivery. Studies reveal that the time of pregnancy affects the chance of having a stillbirth due to exposure to air pollution during pregnancy. There are limited and conflicting studies currently available on the link between exposure to air pollution and stillbirth. On the other hand, other research has demonstrated that throughout pregnancy, exposure concentrations of CO and PM_2.5_ consistently exhibit favorable connection with stillbirth (29, 30).

### 3.1 Association of PM with stillbirth

Studies have shown that exposure to PM during various gestational periods can influence the incidence of stillbirth. Across the entire pregnancy, each 10 μg/m^3^ increase in PM_2.5_ exposure was linked to a 3.4% higher risk of stillbirth (31). During the first trimester, research conducted in China’s coastal regions indicated that every 10 μg/m^3^ increase in PM_2.5_ and PM_10_ exposure corresponded to a 14% (*RR* = 1.14) and 9% (*RR* = 1.09, 95%*CI*: 1.04–1.14) higher risk of stillbirth, respectively (29). Similarly, during the second trimester, each 10 μg/m^3^ increase in maternal PM_2.5_ exposure was significantly associated with an 11% higher risk of stillbirth (29). In the third trimester, every 10 μg/m^3^ rise in PM_10_ exposure was associated with an 8% higher risk of late-pregnancy stillbirth (*aOR* = 1.08, 95%*CI*: 1.04–1.11) (30), while a cohort study from Ohio demonstrated a significant association between elevated PM_2.5_ exposure during the third trimester and increased stillbirth risk (30) (Table 2).

**Table 2:** The *aOR* value and *95%CI* of stillbirth increaseed in pollutant concentration during late pregnancy.

Pollutant concentrations	Air pollution	Analysis city	*aOR*	Outcome	95%*CI*	Risk increase rate	Reference
10 μg/m^3^	PM_10_	Wuhan	1.08	Stillbirth	1.04–1.11	8%	Yang et al. (30)
10 μg/m^3^	NO_2_	Wuhan	1.13	Stillbirth	1.07–1.21	13%	Yang et al. (30)
10 μg/m^3^	SO_2_	Wuhan	1.26	Stillbirth	1.16–1.35	26%	Yang et al. (30)
10 μg/m^3^	PM_2.5_	Ohio	1.42	Stillbirth	1.06–1.91	42%	DeFranco et al. (61)
10 μg/m^3^	O_3_	Wuhan	1.07	Stillbirth	1.05–1.09	7.0%	Tan et al. (31)
10 μg/m^3^	CO	Wuhan	1.01	Stillbirth s	1.00–1.03	1%	Tan et al. (31)

### 3.2 Association of O_3_ with stillbirth

O₃ exposure has been consistently associated with trimester-specific risks of stillbirth across multiple studies. In China’s coastal regions, prospective birth cohort data indicated that a 10 μg/m^3^ increase in first-trimester O₃ exposure was linked to a 5% higher incidence of stillbirth (*RR* = 1.05, 95%*CI*: 1.01–1.09), while third-trimester exposure corresponded to a 4% increased risk (*RR* = 1.04, 95%*CI*: 1.00–1.08) (29). In London, second-trimester O₃ exposure was notably associated with a 22% higher risk of asphyxia-related stillbirth (*OR* = 1.22, 95%*CI*: 1.01–1.43), a 17% increase in all-cause stillbirth (*OR* = 1.17, 95%*CI*: 1.07–1.27), and a 20% elevated neonatal mortality rate (*OR* = 1.20, 95%*CI*: 1.09–1.32) (32), findings consistent with earlier research linking first-trimester O₃ exposure to stillbirth (33). The Wuhan cohort study (n = 509,057 mother-infant pairs; January 2011–September 2017) corroborated these patterns, demonstrating through logistic regression modeling that each 10 μg/m^3^ increase in first-trimester O₃ exposure was associated with a 7.0% higher stillbirth risk (*OR* = 1.07, 95%*CI*: 1.05–1.09), and a 4.0% increased risk per 10 μg/m^3^ increase in third-trimester exposure (delivery window: 28 weeks prior) (31).

### 3.3 Association of others main air pollution with stillbirth

Nitrogen dioxide (NO₂) exposure during late pregnancy consistently elevates stillbirth risk, as evidenced by two Chinese studies: In Wuhan, each 10 μg/m^3^ increase was associated with 13% higher risk (*aOR* = 1.13, 95%*CI*: 1.07–1.21) (30), while Eastern coastal regions showed a 20% increase (*RR* = 1.20, 95%*CI*: 1.09–1.32) (29). For sulfur dioxide (SO₂), two Wuhan-based studies confirmed late-pregnancy exposure effects: one reported 26% elevated risk (*aOR* = 1.26, 95%*CI*: 1.16–1.35) (30), and another found 5.9% increased risk per 10 μg/m^3^ (*OR* = 1.059) (31). Regarding carbon monoxide (CO), early-pregnancy exposure increments of 100 μg/m^3^ raised stillbirth risk by 1.0% (*OR* = 1.01, 95%*CI*: 1.00–1.03) (31).

Due to the differences in time, region, subjects, variable collection, pollutant exposure measurement, and other factors, the above research results may be inconsistent. Air pollution raises the chance of stillbirth, according to many study findings, but since all pregnant women are at risk and stillbirth can inflict considerable physical and psychological stress, there is likely to be a significant overall influence on the stillbirth rate.

## 4 Association of air pollution with spontaneous abortion

Spontaneous abortion (SAB) is when a fetus dies at less than 20 weeks. Currently, pertinent research on how air pollution affects spontaneous abortion mostly focuses on the effect of PM on abortion, and the results are relatively consistent.

Exposure was found to be linked to an increased risk of SAB in a crossover analysis of cases in the United States. Higher rates of SAB were linked to increases in PM_10_ (3.9 μg/m^3^), PM_2.5_, 10 (2.3 μg/m^3^), and PM_2.5_ (2.0 μg/m^3^) in the year before pregnancy 1.12 (95%*CI*: 1.06–1.19), 1.09 (95%*CI*: 1.03–1.14), and 1.10 (95%*CI*: 1.04–1.17), respectively (34). Studies carried out in Jiangsu Province, China, revealed that the likelihood of a spontaneous miscarriage rose by 43.3% for every 10 μg/m^3^ increase in the average PM_2.5_ concentration during pregnancy (35). Xue et al. (36, 37) discovered that exposure to fine particulate matter pollution during pregnancy significantly increased the probability of pregnancy failure. Africa has the highest incidence of abortion worldwide, while South Asia has the highest number of cases. Their epidemiological study focused on these regions. The findings of an additional meta-analysis demonstrated that a 10 μg/m^3^ rise in PM_2.5_ and PM_10_ was linked to a higher risk of SAB, with corresponding combined relative risks of 1.20 (95%*CI*: 1.01 to 1.40) and 1.09 (95%*CI*: 1.02 to 1.15) (38). Numerous studies have shown that PM has a negative impact on miscarriage, so it’s critical to limit PM exposure when pregnant.

## 5 Air pollution’s impact on birth defects

Birth abnormalities have long been associated with environmental issues, including air pollution. Research from the drug withdrawal event in the 1960s to this century on the effects of air pollution on the early growth and development of the fetus, the exchange of nutrients and oxygen with the mother, etc., also demonstrate that air pollution is a risk factor for the fetus’s growth and development (39, 40). Recent research has demonstrated a link between the development of fetal cardiovascular and respiratory disorders and pregnant women’s exposure to air pollution.

### 5.1 Cardiovascular system: congenital heart defects (CHD)

In eastern China, a cross-sectional study discovered a strong link between air pollution and increased CHD in babies born in cities with higher average years of education and in the winter. A 10 μg/m^3^ increase in exposure to PM_2.5_, PM_10_, SO₂, NO₂, CO and O₃ was reported to be linked to an increased risk of CHD. Specifically, O_3_ (*RR* = 1.001, 95%*CI*: 1.000–1.002) in the first trimester, PM_2.5_ (*RR* = 1.025, 95%*CI*: 1.016–1.038) in the third trimester, PM_10_ (*RR* = 1.001, 95%*CI*: 1.000–1.002), and NO_2_ (*RR* = 1.020, 95%*CI*: 1.004–1.036) in the third trimester (Table 3) (41).

**Table 3:** The *RR* value and 95%*CI* of CHD with each increase of 10 μg/m^3^ of pollutant concentration during different pregnancy periods.

Pollutant concentrations	Air pollution	Analysis city	*aOR*	Outcome	95%*CI*	Period	Reference
10 μg/m^3^	PM_2.5_	Hefei	1.081	CHD	1.02–1.146	Early	Zhang et al. (42)
10 μg/m^3^	PM_2.5_	Hefei	1.034	CHD	1.007–1.062	Middle	Zhang et al. (42)
10 μg/m^3^	PM_2.5_	Hefei	1.528	CHD	1.085–2.153	Late	Zhang et al. (42)
10 μg/m^3^	O_3_	Eastern China	1.001	CHD	1.000–1.002	Early	Li et al. (41)
10 μg/m^3^	PM_2.5_	Eastern China	1.025	CHD	1.016–1.038	Late	Li et al. (41)
10 μg/m^3^	PM_10_	Eastern Chain	1.001	CHD	1.000–1.002	Late	Li et al. (41)
10 μg/m^3^	NO_2_	Eastern Chain	1.020	CHD	1.004–1.036	Late	Li et al. (41)
10 μg/m^3^	NO_2_	Suzhou	1.318	CHD	1.210–1.435	Early	Sun et al. (45)
10 μg/m^3^	CO	Henan	1.066	CHD	1.10–1.125	Early	Zhang et al. (46)
10 μg/m^3^	CO	Henan	1.065	CHD	1.012–1.122	Middle	Zhang et al. (46)

In Hefei, China, Zhang et al.’s study (42) revealed that throughout the 20–26-week gestation period, the risk of CHD rose with each 10 μg/m^3^ increase in PM_2.5_. The 22nd week (*RR* = 1.034, 95%*CI*: 1.007–1.062), the 0th week (*RR* = 1.081, 95%*CI*: 1.02–1.146), the 37th week (*RR* = 1.528, 95%*CI*: 1.085–2.153), and the 40th week (*RR* = 1.171, 95%*CI*: 1.006–1.364) were the most affected by the four pollutants, namely PM_2.5_, PM_10_, SO_2_, and NO_2_. A Guangdong, China study used logistic regression analysis to demonstrate that early prenatal exposure to all air pollutants by the mother was linked to a higher risk of congestive heart failure. A 1.09-fold increase in the risk of CHD was linked to an increase in the PM_10_ quartile interval (13.3 μg/m^3^) (43).

Researchers discovered that exposure to high concentrations of SO_2_ in early pregnancy raised the risk of CHD by 64%, while exposure to high concentrations of PM_10_ in early pregnancy increased the risk of CHD by 46% using a case–control analysis of infants born in Shenyang between 2011 and 2015. The study did not find a meaningful association between preconception and pregnancy NO_2_ exposure and an increased risk of CHD (44). However, a subsequent study in Suzhou, China, analyzed the prevalence of CHD in children born between 2015 and 2019, and demonstrated a substantial correlation between early pregnancy maternal NO_2_ exposure and CHD (*aOR* = 1.318, *95%CI*: 1.210–1.435) (45). The difference between the two research results may be due to the inconsistency of birth time and the living environment of pregnant women in the case samples.

Fetuses from single live births enrolled in Henan Province between 2013 and 2018 were subjected to a case–control analysis, which revealed that exposure to CO in the first and second trimesters increased the overall risk of CHD, with *aOR* and 95% *CI* of 1.066 (1.010 to 1.125) and 1.065 (1.012 to 1.122), respectively. Additionally, the study demonstrated that the first 6 weeks of pregnancy were the primary window of sensitivity to air pollution and ASD (46).

### 5.2 Respiratory system: lung development

Research has found an association between air pollution exposure during pregnancy and early life and impaired lung development, reduced pulmonary function and respiratory conditions in childhood, such as asthma and wheezing. These effects may persist in adulthood (47). Air pollution has also been connected to decreased lung function in infancy and children, an increase in respiratory symptoms, and the onset of childhood asthma. It may also indirectly affect lung development, resulting in premature birth or immune system disruptions (48).

In the TIDES and CANDLE cohorts, there is a 29% increased risk of early childhood asthma (mean 4.3 years, standard deviation 0.5 years) for every 2 μg/m^3^ increase in PM_2.5_ exposure during the cystic phase (24–36 weeks of gestation) (49). This suggests that fetal lung development may be particularly sensitive to the developmental toxicity of PM_2.5_ later in life. Increased exposure to household air pollution during pregnancy was linked to lower lung function in newborns, according to another study. The time from peak tidal expiratory flow to expiratory time decreased (*β* = −0.004, *p = 0.01*), the respiratory rate rose (β = 0.28, *p = 0.01*), and minute ventilation increased (β = 7.21, *p = 0.05*) with each 1 ppm increase in mean prenatal CO. At the same time, impaired lung function in infants may increase the risk of pneumonia in the first year of life (50).

More and more evidence shows that the long-term malignant accumulation of air pollutants may become a hidden danger of birth defects (51), and living in an environment with heavy air pollution should be avoided during pregnancy.

## 6 Possible mechanisms by which air pollution affects ABO

Maternal exposure to air pollution indirectly harms fetal development through placental transfer (28). Pregnant women are especially vulnerable to the effects of air pollution because their oxygen consumption is 15–20% higher than that of non-pregnant adults, and their breathing volume per minute is 30–40% higher (52). Further studies have found that air pollutants can cross the placental barrier and thus adversely affect the fetus (53). Meanwhile, cells are characterized by rapid replication and differentiation during embryonic development, they are more sensitive to external exposure events (54). Therefore, pollutants such as NO_2_, SO_2_ and O_3_ will cause irreversible damage to divided cells (55) and damage to embryos at key stages of development.

Air pollution exposure can damage the microstructure of germ cells. Wilhelm et al.’s research findings (56) demonstrated that SO_2_ and its derivatives had harmful effects on the microstructure and functionality of germ cells as well as specific impacts on the growth of germ cells and embryos *in vivo*. Therefore, genetics could be utilized to elucidate the substantial impact of air pollution exposure on the risk of stillbirths, fetal deaths and the occurrence of birth malformations. According to Behlen et al. (57), air pollution may have a negative impact on a woman’s pregnancy by altering the placenta’s gene expression. Because of its effects on the expression of genes related to lipid metabolism, inflammation, and antioxidant defense, air pollution increases the risk of pre-eclampsia and other unfavorable pregnancy outcomes. These results shed new light on the connection between poor birth outcomes and air pollution. Furthermore, air pollution will impact the immune system and the metabolism of maternal hormones, which will worsen the consequences of pregnancy (58).

Although the exact mechanism by which exposure to air pollution worsens unfavorable birth outcomes is yet unknown, the harmful effects of carbon monoxide (CO) on the developing fetus by affecting fetal oxygen delivery are well recognized. Specifically, while fetal hemoglobin has a higher binding compatibility with CO than adult hemoglobin, CO will cross the placental barrier and more seriously affect the oxygen delivery of the fetus (59), leading to premature delivery, stillbirth, birth defects, and other unfavorable outcomes. Meanwhile, CO exposure will also reduce the oxygen-carrying capacity of maternal hemoglobin.

## 7 Review and prospects

**Figure 1:**
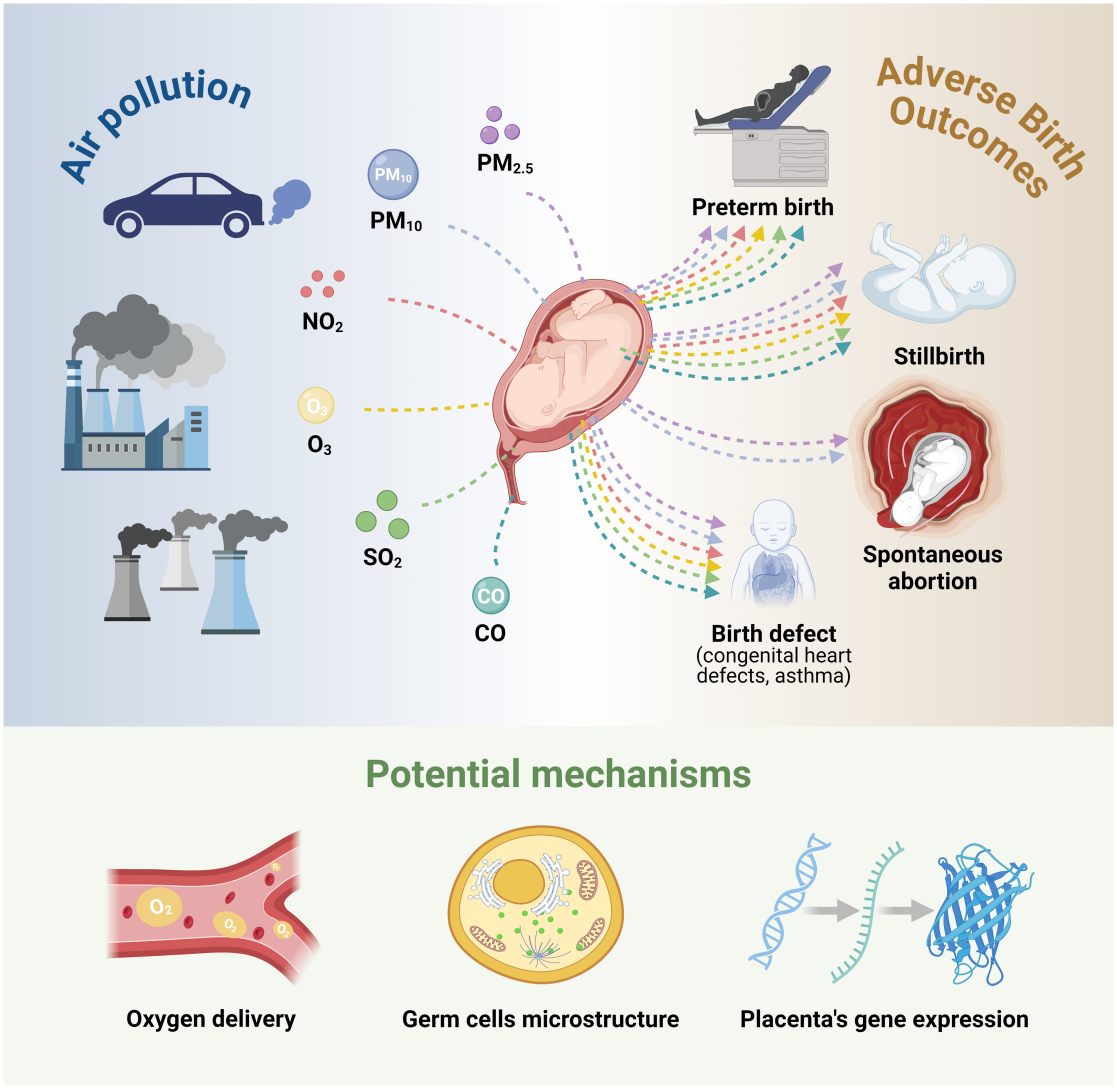
The logic map of air pollution’s toll on adverse birth outcomes. Relationships between air pollutants, adverse birth outcomes, and potential biological mechanisms. Proposed pathways include: (1) disruption of fetal oxygen delivery, (2) alteration of germ cell microstructure, and (3) pollution-induced changes in placental gene expression.

The link between major air pollutants PM, NO_2_, O_3_, SO_2_, and CO unfavorable birth outcomes is the main topic of this research. Air pollution can result in unfavorable birth outcomes such as lung dysplasia, immune system development disorder, and improper cardiovascular system development, in addition to preterm birth, stillbirth, and spontaneous abortion (Figure 1). Although the exact mechanism by which air pollution leads to adverse birth outcomes is not yet clear and may involve nitrosation/oxidative stress, inflammation, endocrine disorders, epigenetic changes, and vascular dysregulation of the maternal fetal unit (60), the harmful effects of CO, NO_2_, SO_2_, and O_3_ on developing fetuses have been identified. To prevent or lessen the negative effects of air pollution on pregnancy outcomes, fetal development, child growth, and maternal health, as well as to provide a scientific basis for the formulation of air quality standards and management policies, drug intervention for pregnant women in areas with severe air pollution can be studied from the perspective of molecular mechanisms in the future. Furthermore, despite China’s best efforts to reduce air pollution in recent years, the country’s air quality has rapidly improved. However, because of China’s massive population base and unequal social development, many pregnant women are still exposed to air pollution. It is yet unknown, nevertheless, how exposure to air pollution affects the likelihood of pregnancy failure in particular communities. To provide theoretical support for the prevention and control of air pollution and the health of special populations, more prospective, multi-center, large-sample cohort studies and multivariate analyses are required in the future to investigate the effects of various pollutants on pregnancy outcomes in different window periods. Meanwhile, in future studies, emphasis should be placed on the exposure time and cumulative effects of various pollutants.
